# Protocol: mixed-methods study to evaluate implementation, enforcement, and outcomes of U.S. state laws intended to curb high-risk opioid prescribing

**DOI:** 10.1186/s13012-018-0719-8

**Published:** 2018-02-26

**Authors:** Emma E. McGinty, Elizabeth A. Stuart, G. Caleb Alexander, Colleen L. Barry, Mark C. Bicket, Lainie Rutkow

**Affiliations:** Baltimore, USA

**Keywords:** Opioid, Law, Synthetic control, Mixed-methods

## Abstract

**Background:**

The U.S. opioid epidemic has been driven by the high volume of opioids prescribed by healthcare providers. U.S. states have recently enacted four types of laws designed to curb high-risk prescribing practices, such as high-dose and long-term opioid prescribing, associated with opioid-related mortality: (1) mandatory Prescription Drug Monitoring Program (PDMP) enrollment laws, which require prescribers to enroll in their state’s PDMP, an electronic database of patients’ controlled substance prescriptions, (2) mandatory PDMP query laws, which require prescribers to query the PDMP prior to prescribing an opioid, (3) opioid prescribing cap laws, which limit the dose and/or duration of opioid prescriptions, and (4) pill mill laws, which strictly regulate pain clinics to prevent nonmedical opioid prescribing. Some pain experts have expressed concern that these laws could negatively affect pain management among patients with chronic non-cancer pain. This paper describes the protocol for a mixed-methods study analyzing the independent effects of these four types of laws on opioid prescribing patterns and chronic non-cancer pain treatment, accounting for variation in implementation and enforcement of laws across states.

**Methods:**

Many states have enacted multiple opioid prescribing laws at or around the same time. To overcome this issue, our study focuses on 18 treatment states that each enacted a single law of interest, and no other potentially confounding laws, over a 4-year period (2 years pre-/post-law). Qualitative interviews with key leaders in each of the 18 treatment states will characterize the timing, scope, and strength of each state law’s implementation and enforcement. This information will inform the design and interpretation of synthetic control models analyzing the effects of each of the two types of laws on two sets of outcomes: measures of (1) high-risk opioid prescribing and (2) non-opioid treatments for chronic non-cancer pain.

**Discussion:**

Study of mandatory PDMP enrollment, mandatory PDMP query, opioid prescribing cap, and pill mill laws is timely given a dynamic policy environment in which numerous states pass, revise, implement, and enforce varied laws to address opioid prescribing each year. Findings will inform enactment, implementation, and enforcement of these laws in additional states.

## Background

The U.S. opioid epidemic was associated with over 65,000 opioid overdose deaths [1, 2] and decreased U.S. life expectancy during 2015–2016 [3]. This epidemic has been driven in large part by the high volume of opioids prescribed by healthcare providers [4–7]. Increases in opioid prescribing beginning in the late 1990s led to parallel increases in opioid overdose deaths, which quadrupled from 1999 to 2015 [8–10]. In recent years, growing rates of prescription opioid addiction have contribued to increased rates of heroin and synthetic opioid (e.g. fentanyl) use [11], which has further driven the upward trend in opioid overdose deaths [1]. At the same time, an estimated 25 million U.S. adults experience daily chronic pain [12], and prominent pain medicine clinicians and advocates have expressed concern that efforts to curb opioid prescribing could negatively impact chronic pain management [13, 14].

Over the past decade, U.S. states have enacted multiple types of laws designed to curb high-risk opioid prescribing practices associated with opioid misuse, dependence, and mortality, including high-dose opioid prescribing, long-term opioid prescribing for acute pain, and overlapping opioid and benzodiazepine prescriptions [15]. Most recently, states have focused on four types of laws:

1. *Mandatory prescription drug monitoring program (PDMP) enrollment laws* require healthcare providers to enroll in—and thereby gain access to—their state’s PDMP, an electronic database prescribers can query to learn about patients’ controlled substance prescription drug history. For example, Colorado has a mandatory PDMP enrollment law that requires every practitioner with a current federal Drug Enforcement Administration controlled substances registration to enroll with the state’s PDMP.
2. *Mandatory PDMP query laws* require providers to check their state’s PDMP prior to prescribing opioids. For example, Pennsylvania has a law requiring that providers query the PDMP at the outset of a patient’s opioid treatment plan or if the prescriber believes the patient is diverting or abusing drugs.
3. *Opioid prescribing cap laws* limit the days’ supply and/or dose of prescribed opioids. For example, New York State has a law imposing a 7-day limit on initial opioid prescriptions for acute pain.
4. *“Pill mill” laws* strictly regulate pain management clinics to prevent rogue clinics (“pill mills”) from issuing opioid prescriptions without medical indication. For example, Texas law requires pain clinics to be owned by physicians with unrestricted licenses and to be certified with and undergo inspections by the state.

As of January 1, 2017, the cut point for inclusion in the study described in this protocol, 20 states had mandatory PDMP enrollment laws,^1^ 20 states had mandatory PDMP query laws,^2^ seven states had opioid prescribing cap laws,^3^ and 11 states had pill mill laws.^4^ Despite enactment in multiple states, the independent effects of these four types of state laws on opioid prescribing patterns remain poorly understood. Limited prior studies evaluating these laws are hampered by two primary methodological concerns.

First, some states have implemented multiple laws of interest at or around the same time, making it difficult to disentangle the independent effects of different types of laws. For example, one study found that simultaneously implementing pill mill and mandatory PDMP query laws reduced opioid prescribing but could not separate the independent effects of the two laws [16].

Second, prior studies have not assessed implementation and enforcement of the laws of interest [16–21]. State laws with weak implementation and/or enforcement are unlikely to reduce high-risk opioid prescribing, but previous quantitative studies have not incorporated implementation or enforcement data into models estimating laws’ effects on opioid prescribing patterns. For mandatory PDMP enrollment and query laws, key implementation considerations include awareness of and compliance with the law, quality of Health IT infrastructure (prescribers are more likely to adhere to PDMP enrollment and query laws if PDMP databases are easy to access and search), and completeness of the prescription data included in the PDMP [22, 23]. Key enforcement considerations include whether and how states enforce mandatory PDMP enrollment and query laws [22, 23]. Do states have mechanisms for determining if prescribers fail to enroll/query? Are audits conducted? Are there penalties if lack of adherence is identified? Are penalties actively enforced? For opioid prescribing cap laws, the development of systems to track compliance with prescribing limits—potentially through PDMPs—may be a key component of implementation and enforcement. Common pill mill law provisions include requirements that clinics are owned by physicians with unrestricted licenses, limits on patient/prescriber ratio, and a mandatory annual licensing process [24]. States’ capacity to enforce these laws through inspections, audits, and penalties likely plays a key role in how such laws influence high-risk opioid prescribing [24]. Partnerships with law enforcement may also play an important role in pill mill law implementation and enforcement; in Florida, law enforcement played a lead role in implementation of that state’s pill mill law by identifying and closing pill mills [25]. For all laws, education campaigns targeting implementers/enforcers, clear designation of responsibility for enforcement, and allocation of resources for implementation and enforcement are key considerations.

This mixed-methods study is designed to overcome these methodological challenges through use of a combination of legal research, qualitative interviews with key implementation and enforcement stakeholders, and synthetic control analyses of secondary administrative claims data. The overarching goals of the study are to characterize the implementation and enforcement of the four types of state laws of interest and to evaluate the independent effects of those laws, accounting for variation in implementation and enforcement across states, on two primary sets of outcomes: (1) high-risk opioid prescribing patterns and (2) treatment of chronic non-cancer pain. All phases of the study will be supported by an advisory board comprised of key stakeholders including leaders from the fields of pain and addiction medicine and drug policy.

## Methods

### Study aims and hypotheses

Our study will focus on 18 “treatment states” that each enacted one of the four types of laws of interest, but no other potentially confounding laws that could affect opioid prescribing, during a 4-year study period (2 years pre-/post-law). These 18 treatment states were identified through legal research, as described in the “Study design” section below. The study’s three specific aims and the hypotheses for its quantitative aims (2–3) are as follows:

- Aim 1

Study Aim 1 is to characterize the implementation and enforcement of state laws designed to curb high-risk opioid prescribing. In each of the 18 treatment states, we will conduct semi-structured interviews with 5–10 key stakeholders, including leaders of state healthcare societies and state regulators, including PDMP, health department, and licensing board administrators. The interviews will focus on characterizing the implementation (e.g., educational campaigns to increase prescribers’ awareness of the laws) and enforcement (e.g., routine inspections of pain clinics) of the laws of the interest. The results of the Aim 1 qualitative interviews with stakeholders in the 18 treatment states will inform the design and interpretation of the Aims 2 and 3 quantitative analyses.

- Aim 2

Study Aim 2 is to evaluate the independent effects of state laws designed to curb high-risk opioid prescribing on opioid prescribing patterns. We will use synthetic control analyses to evaluate the four types of state laws’ effects on high-risk opioid prescribing. We expect each type of law to independently reduce the overall volume of opioid prescribing, average dose per opioid prescription, and average days’ supply of prescribed opioids. Because PDMP use allows prescribers to identify patients’ controlled substance prescription history, including prescriptions from other clinicians, we hypothesize that mandatory PDMP enrollment and mandatory PDMP query laws will also reduce overlapping opioid and benzodiazepine prescriptions and doctor shopping, or the percent of patients simultaneously receiving opioid prescriptions from multiple doctors. For all laws studied, we expect those with strong implementation and enforcement—as identified through Aim 1 interviews—to have the largest effects on high-risk opioid prescribing practices.

- Aim 3

Study Aim 3 is to evaluate the independent effects of state laws designed to curb high-risk opioid prescribing on the treatment of chronic non-cancer pain. The state laws of interest do not apply to chronic cancer pain, for which opioid therapy is widely accepted. Again, using synthetic control analyses, we will examine laws’ effects on pain treatment for individuals diagnosed with three common pain conditions: lower back pain, headache, and fibromyalgia. Recent clinical guidelines and evidence summaries conclude that the risks of opioid treatment often outweigh the benefits for these conditions and recommend alternative non-opioid pain treatments [26–31]. We expect each type of law to independently reduce high-risk opioid prescribing among individuals with these three conditions in the same manner as hypothesized in Aim 2. We also expect that reductions in opioid prescribing among these patients will be accompanied by increases in non-opioid pharmacologic (e.g., anticonvulsants, NSAIDs) and non-pharmacologic (e.g., steroid injections, physical therapy) pain treatments. For all laws studied, we expect those with strong implementation and enforcement—as identified through Aim 1 interviews—to have the largest effects on high-risk opioid prescribing practices.

### Study design

The primary quantitative analysis approach to estimate the effects of the laws will use a synthetic control design [32]. Briefly, this method compares outcomes after a law is enacted in a single-treatment state to outcomes in a weighted combination of comparison states, or the “synthetic control.” In other words, to evaluate the effects of a law enacted in Ohio, this method combines data from eligible comparison states to create a “synthetic Ohio” with similar demographic characteristics and pre-law trends in the outcome of interest; the synthetic control is designed to approximate the treatment state’s post-law trends in the outcome of interest had the law of interest not been enacted (i.e., the counterfactual). Treatment and control pool states selected for our study are shown in Table 1.

**Table 1:** Treatment and comparison states

Treatment state, effective date	Study period	Eligible comparison states (“control pool”) *See footnotes for opioid prescribing legal environment criteria used to select control pool states*
Mandatory PDMP enrollment law		
CO, 1/1/15	1/1/13–12/31/16	IA, KS, MI, MT, NE, ND, OR, SC, SD, WY^1^
ID, 7/1/14	7/1/12–6/30/16	CA, IA, KS, MI, MN, MT, NE, ND, OR, SC, SD, WY^1^
IL, 9/9/15	9/9/13–8/8/17	IA, KS, MI, MT, NE, ND, OR, SC, SD, WY^1^
NM, 1/1/11	1/1/09–12/31/12	AL, CO, CT, ID, IN, IA, MA, MI, MN, NV, NY, ND, OK, PA, SC, TN, VA, WY^1^
UT, 9/30/10	9/30/08–9/29/12	AL, CO, ID, IL, IN, MA, MI, MN, NV, NY, OK, PA, RI, SC, VA, WV, WY^1^
Mandatory PDMP query law		
IN, 11/1/14	11/1/12–10/31/16	CA, IA, KS, MI, MN, MT, NE, ND, OR, SC, SD, WY^1^
NY, 8/27/13	8/27/11–8/26/15	CA, CO, IA, KS, MI, MN, NV, NJ, ND, OR, RI, SC, SD, WY^1^
OK, 11/1/15	11/1/13–10/31/17	IA, KS, MI, MT, NE, ND, OR, SC, SD, WY^1^
PA, 6/30/15	6/30/13–6/29/17	IA, KS, MI, MT, NE, ND, OR, SC, SD, WY^1^
VA, 7/1/15	7/1/13–6/30/17	IA, KS, MI, MT, NE, ND, OR, SC, SD, WY^1^
Prescribing cap law		
CT, 7/1/16	7/1/15–6/30/18	IN, NH, NJ, PA, VA, VT^3,4^
NH, 7/1/18	7/1/16–6/31/20	IN, NJ, OK, PA, and VA^3^
NY, 7/1/16	7/1/15–6/30/18	IN, NH, NJ, PA, VA, VT^3,4^
RI, 7/1/16	7/1/15–6/30/18	IN, NH, NJ, PA, VA, VT^3,4^
VT, 7/1/18	7/1/16–6/31/20	IN, NJ, OK, PA, VA^3^
Pill mill law		
MS, 3/1/11	3/1/09–2/28/13	AL, CO, ID, IN, IA, MI, NV, NY, ND, OK, PA, RI, SC, VA, WY^2^
OH, 7/1/2011	7/1/09–6/30/13	AL, CO, ID, IN, IA, MA, MI, NV, NY, ND, OK, PA, RI, SC, VA, WY^2^
TX, 9/1/10	9/1/08–8/31/12	AL, CO, CT, ID, IL, IN, KT, MA, MI, NV, NY, OK, PA, RI, SC, TN, VA, WV, WY^2^

^1^Treatment states transitioned from having a voluntary PDMP law in the pre-law period to a mandatory PDMP enrollment/query law in the post-law period; all treatment/control pool states had voluntary PDMP, physical exam, and doctor shopping laws—and no other laws of interest or potentially confounding laws—in effect for the entire study period

^2^Treatment/control pool states had voluntary PDMP, physical exam, and doctor shopping laws—and no other laws of interest or potentially confounding laws—in effect for the entire study period

^3^Treatment/control pool states had physical exam, doctor shopping, and mandatory PDMP query laws—and no other laws of interest or potentially confounding laws—in effect for the entire study period

^4^Three-year study period (1 year pre-law/2 years post-law). If a 4-year study period is applied, Indiana is the only eligible control pool state

### Identification of treatment states

The synthetic control method is designed to evaluate the effects of a single law in a single treatment state [32]. To make valid causal inferences from this method, the law of interest must be the only law enacted during the study period that could change the outcome under study. For the proposed study, treatment states were defined as states that implemented a single law of interest (mandatory PDMP enrollment, mandatory PDMP query, opioid prescribing cap, or pill mill law)—and no other laws of interest or other potentially confounding state laws that could influence opioid prescribing over a 4-year period, 2 years pre-/post-law. To identify states meeting this definition, we accounted for potentially confounding laws, which included (1) voluntary PDMP laws, which establish a PDMP but do not require prescriber enrollment or query, (2) physical exam laws, which require a physical examination before opioids are prescribed, (3) doctor shopping laws, which prohibit individuals from seeking overlapping opioid prescriptions from multiple providers, and (4) mandatory pharmacy ID laws, which require identification when picking up an opioid at the pharmacy in order to prevent individuals from fraudulently obtaining another individual’s prescription. We considered these laws as potential confounders rather than laws of interest for two reasons. First, voluntary PDMP, physical exam, and doctor shopping laws have now been enacted in all states, so studying the effects of these laws will not inform enactment in additional states. Second, our study aims to evaluate state laws designed to curb high-risk opioid prescribing by healthcare providers. Doctor shopping and mandatory pharmacy ID laws focus on patient, rather than provider, behavior.

### Identification of comparison states

In the synthetic control method, each treatment state has its own group of eligible “control pool” states [32]. States must meet two criteria for inclusion in the control pool. First, they must have no changes in laws that could influence opioid prescribing outcomes (either laws of interest or potentially confounding laws) during the study period. Second, with the exception of the law of interest in the treatment state, the control pool states must have the same opioid prescribing laws as the treatment state for the entire study period. For example, consider the treatment state Ohio, which implemented a pill mill law on July 1, 2011. For the entire July 1, 2009, to June 30, 2013, study period, Ohio had voluntary PDMP, physical exam, and doctor shopping laws and no other laws of interest or potentially confounding laws, except the enactment of the July 1, 2011, pill mill law. Eligible control pool states therefore had voluntary PDMP, physical exam, and doctor shopping laws and no other laws of interest or potentially confounding laws in place for the same study period.

### Study period

The 4-year study period is unique to each treatment state. In three states enacting prescribing cap laws, the study period was truncated from 4 to 3 years (1 year pre- and 2 years post-law) to allow for an adequate number of control pool states. If states implemented multiple laws of interest with sufficient time between laws, they could be included in the study as treatment states more than once.

### Legal research to identify treatment and control pool states

To identify treatment and control pool states meeting the selection criteria described above, we used standard legal research and legislative history techniques [33]. For each of the laws of interest, we used standardized search terms within the Westlaw legal database. Within this database, we were able to search the full text of every state’s codified laws, session laws, bills, and regulations. Once we determined the presence of relevant laws, we identified their effective date. When the effective date was not included in the codified law, we consulted session laws, regulatory materials, or materials posted on government websites.

For quality control purposes, we compared our findings with publicly available materials compiled by organizations including the PDMP Training and Technical Assistance Center and the National Alliance for Model State Drug Laws. When we found inconsistencies between our results and these materials, we consulted the text of the relevant law as well as members of our advisory board. We then used our findings to identify the 18 treatment states and affiliated control pools (Table 1).

### Data sources

#### Qualitative interview data

Aim 1 qualitative data will be collected through semi-structured interviews with key implementation and enforcement leaders using a common interview guide, with questions in three domains: perceptions of the law of interest (e.g., perceptions of how the relevant law has affected opioid prescribing), implementation of the law (e.g., challenges and delays in implementation, implementation successes), and enforcement of the law (e.g., penalties for failure to comply with the law, challenges, and delays in enforcement activities; enforcement successes). All interviews will be conducted by a single master’s level research assistant trained in qualitative interviewing techniques. Interviews will be conducted by telephone, recorded, and transcribed. The interview guide will be developed by the study team and refined based on advisory board members’ feedback. To promote transparency of our planned qualitative research, Table 2 presents qualitative research design aspects within the COnsolidated criteria for REporting Qualitative (COREQ) studies framework [34], including additional details regarding interview guide development and data collection.

**Table 2:** Qualitative study design

*Domain 1: Research team and reflexivity*	
Personal characteristics	
1. ^1^ Interviewer/facilitator	All interviews will be conducted by the same member of the study team
2. Credentials	The interviewer will be a masters-level trained research assistant
3. Occupation	The interviewer will be employed full-time as a research assistant
4. Gender	The interviewer will be female
5. Experience and training	The interviewer will have experience participating in qualitative research studies and will be supervised by study co-PIs with extensive training and experience conducting qualitative research
Relationship with participants	
6. Relationship established	Potential interviewees will be contacted with a standardized recruitment email to introduce the study and the interviewer and to request their participation
7. Participant knowledge of the interviewer	The recruitment email will explain the study goals and why the interviewer is interested in conducting this research. This information will be reviewed at the start of each interview
8. Interviewer characteristics	The recruitment email will provide information about the research team, including the interviewer. This information will be reviewed at the start of each interview
*Domain 2: Study design*	
Theoretical framework	
9. Methodological orientation and theory	The qualitative portion of the study will use a content analysis approach
Participant selection	
10. Sampling	Potential interviewees will be selected based on their legally established responsibilities relative to the state law(s) of interest
11. Method of approach	Potential interviewees will be approached with a standardized recruitment email
12. Sample size	We anticipate conducting 5 to 10 interviews in each of 18 treatment states
13. Non-participation	We will document any reasons provided by those who decline to participate as well as any individuals who do not respond to our recruitment email
Setting	
14. Setting of data collection	Data will be collected via interviews conducted by telephone
15. Presence of non-participants	We anticipate that the interviewer and interviewee will be the only individuals present
16. Description of sample	The sample will include key implementation leaders for the law(s) of interest in each of 18 treatment states
Data collection	
17. Interview guide	The interview guide will be developed by the study team and shared with an advisory board for feedback. It will be pilot-tested and refined before data collection begins
18. Repeat interviews	We do not anticipate conducting repeat interviews
19. Audio/visual recording	Once permission is granted, interviews will be audio recorded
20. Field notes	The interviewer will draft summary notes immediately after concluding each interview
21. Duration	We anticipate that interviews will last no more than 30 min
22. Data saturation	The study team will convene on a regular basis to review interview data and determine when data saturation is reached
23. Transcripts returned	We do not plan on returning transcripts to interviewees. Based on the straightforward nature of our questions and prior research with similar types of interviewees, we do not anticipate that this will be necessary
*Domain 3: Analysis and findings*	
Data analysis	
24. Number of data coders	We plan to have two coders pilot a sub-sample of transcripts. Once discrepancies are resolved and the codebook is finalized, the full set of transcripts will be coded by one individual
25. Description of the coding tree	We plan to develop a coding tree (i.e., codebook) based on a review of the literature, a priori knowledge within the study team, and summary notes from interviews. We will also share a draft codebook with our advisory board for feedback
26. Derivation of themes	Themes will be derived once data have been coded. Preliminary themes may be identified based on discussions with the interviewer and review of field notes
27. Software	We plan to use NVivo qualitative research software
28. Participant checking	A bulleted list of key findings will be shared with participants once data have been coded and analyzed
Reporting	
29. Quotations presented	Quotations from interviews will be used to present findings, and they will be accompanied by an interviewee identification number
30. Data and findings consistent	Our planned use of quotations will allow for assessment of consistency between our data and findings. We will also create supplemental tables with additional quotations to share as much information as possible when presenting our findings
31. Clarity of major themes	We plan to use sub-headings listing our major themes to promote clarity when writing up our findings
32. Clarify of minor themes	We plan to provide quotations from interviewees who raised minor themes or shared information contrary to findings of our major themes

#### Administrative claims data

Aim 2 and 3 quantitative analyses will use IQVIA prescription and outpatient claims data [35]. A key strength of the IQVIA data is that it includes cash-paid services in addition to services paid by insurers. Using an electronic portal linked to insurance billing and practice management software, pharmacies and outpatient practices transmit claims to IQVIA on a daily basis. The IQVIA data include data from all 50 states. The prescription claims data capture approximately 88% of retail prescriptions, and the outpatient claims data capture approximately 60% of U.S. outpatient clinics and individual/group practices. These two data sources will be linked together using a unique patient identifier created by IQVIA. The prescription claims data includes information on product name, form, strength, and quantity, as well as the date dispensed. The outpatient claims data includes ICD-9/ICD-10 diagnosis codes, CPT, HCPCS, ICD-9 and ICD-10 procedure codes, and date of service. Both data sets include state identifiers; patient date of birth, gender, and the date the patient first appeared in the IQVIA data; a unique provider identifier and information on provider specialty and location; and payment information including payment type (cash/insurance) and name of payer (e.g., Medicaid, Medicare, Aetna).

### Study sample

The Aim 1 study sample will include key implementation leaders in each treatment state, where we will begin by interviewing the individual with primary responsibility for implementation and enforcement of the law of interest, as established by the relevant statute. Additional interviewees will be identified through purposive snowball sampling [36] and in conjunction with our advisory board. Interviews will be conducted until no interviewees are providing new information (data saturation) [37]. Interviewees will receive a standard recruitment email explaining study goals and inviting them to participate in the study. We plan to conduct 5–10 leader interviews in each of the 18 treatment states. See Table 2 for additional sample selection details.

The Aim 2 study sample is a continuous cohort of individuals who are present in the IQVIA data and did not move out of treatment/control pool states during the entire 4-year study period. The sample includes patients of all ages who received one or more opioid prescriptions prescribed by a healthcare provider located in the treatment or control pool states during the study periods. Opioid prescriptions will be identified using Uniform System of Classification (USC) codes.

The Aim 3 study sample is a continuous cohort of individuals diagnosed with lower back pain, headache, or fibromyalgia who received one or more opioid prescriptions from healthcare providers located in the treatment and control pool states during the study periods. We will identify sample patients using a comprehensive set of ICD-9 and ICD-10 diagnosis codes. Patients in the IQVIA outpatient claims data who have at least two claims with one of these codes listed as the primary diagnosis will be included.

### Measures

Aim 1 qualitative interviews will characterize implementation and enforcement of the law of interest in each of the 18 treatment states. For example, interview findings might identify delays in implementation of a law or identify states where laws were not enforced. In Aim 2 and 3 synthetic control analysis, the data will be set up at the state-month level and the independent variable is a dichotomous indicator of the law of interest in the treatment state that will “turn on” (from 0 to 1) in the first full month the law is implemented. In Aim 2, dependent variables include measures of high-risk opioid prescribing. In Aim 3, the dependent variables include measures of high-risk opioid prescribing as well as measures of receipt of non-opioid pharmacologic and non-pharmacologic pain treatments.

#### High-risk opioid prescribing

All measures of high-risk opioid prescribing will be calculated using mean morphine milligram equivalents (MMEs) [27]. MMEs standardize opioid prescriptions and account for differences in molecules and the quantity and strength of opioids dispensed. Measures of high-risk opioid prescribing will include:

- Overall volume of opioid prescribing: MME per patient across all opioid prescriptions
- High-dose opioid prescribing: average dose per opioid prescription, average daily dose of opioids across all prescriptions, and indicator of dangerously high daily dose (MME per patient/day> 90 MME)
- Long-term opioid prescribing: average days’ supply per opioid prescription and indicators of long-term (days’ supply > 7 days, days’ supply > 30 days) prescriptions
- Proportion of patients receiving opioids from multiple prescribers (e.g., > 1 and > 3 prescribers)
- Proportion of patients receiving overlapping opioid and benzodiazepine prescriptions

Receipt of non-opioid pharmacologic and non-pharmacologic pain treatments:

Our research team has developed lists of non-opioid pain treatments, as well as the drug (ND and USC) and procedure codes used to identify these treatments in administrative claims data, in prior work. These measures will include the proportion of patients with low back pain, headache, or fibromyalgia receiving:

- NSAIDS, antidepressants, anticonvulsants, topical analgesics, and muscle relaxants used to treat pain
- Cognitive behavioral therapy, biofeedback, ultrasound, physical therapy, occupational therapy, acupuncture, electrical stimulation devices, chiropractic care, spinal neurostimulation, steroid injections, trigger point injections, facet injections, peripheral nerve block injections, and botox injections (some of these treatments are condition-specific, e.g., chiropractic care is used for low back pain and fibromyalgia but not headache)

### Analysis

#### Qualitative analysis

Following each interview, the interviewer will create a document with summary notes to help identify preliminary themes within the data. Interview transcripts will be analyzed using the staged approach to coding. This process begins with general coding and evolves to include more specific coding as data analysis moves forward and researchers develop and refine a working model for the relationships within the data. The research team will create an initial codebook based on review of the literature, a priori knowledge within the study team, and summary notes from interviews. This draft codebook will be reviewed and refined by the advisory board.

Using a randomly selected sub-sample of transcripts, two coders will then pilot the codebook. Any discrepancies that arise will be resolved through a discussion and consensus process with the two coders and a third independent party. If any changes to the coding tree are needed based on the piloting phase, e.g., if additional themes emerge in coders’ reviews of data, then the codebook will be refined. Significant revisions will be shared with the advisory board. The finalized codebook will be applied to all transcripts. Within each transcript, text segments will be organized within qualitative research software and will be analyzed, first descriptively and then according to themes and sub-themes. See Table 2 for additional qualitative analysis details.

Aim 1 qualitative interview results will yield detailed information about implementation and enforcement of the laws of interest. This information will be used to inform development of the quantitative models used for the Aims 2–3 analyses. For example, if we learn that a law’s implementation was delayed by 6 months, we will adjust the study period for that treatment state accordingly. Qualitative interview results will also help us interpret Aims 2–3 synthetic control model results. Findings regarding incomplete implementation or lack of enforcement may explain why a law of interest reduced high-risk opioid prescribing in one state but not another.

#### Synthetic control analyses

Following Abadie et al. [32, 38], we will develop a synthetic control for each treatment state by creating a vector of state-specific weights that minimize the mean squared prediction error (MSPE) between the pre-law trends in the opioid prescribing outcome of interest and covariates in treatment and control pool states. As the goal is to create a synthetic control that parallels the treatment state’s pre-law trends in a specific outcome, a given treatment state will have a unique synthetic control for each outcome of interest. Because each of the 18 treatment states in the study has its own unique synthetic control, we will conduct 18 state-specific analyses for each outcome of interest. Synthetic control fit will be assessed descriptively by graphing trends in opioid prescribing and comparing pre-law outcome and covariate values in each treatment state and its synthetic control, with similar values indicating good fit. We will also examine MSPE; smaller MSPE indicates better fit.

Laws’ effects on outcomes will be measured as the post-law difference in outcomes in a treatment state versus its synthetic control, calculated as the sum of the difference in outcomes in each of the 24 post-law months in the study period. We will conduct a permutation-based test, similar to the Fisher exact test, in which the synthetic control analysis conducted for a given treatment state is repeated for all states in the control pool (i.e., by creating a synthetic control, using other states in the same control pool, for each control state). To assess statistical significance, we will calculate the proportion of control pool states with an estimated post-law “effect” in the outcome of interest that is as or more extreme than the estimated post-law effect in the treatment state; this proportion is akin to the *P* value. We will assess the robustness of results by calculating this proportion four times, using different groups of control pool states with varying degrees of synthetic control fit: (1) all control pool states and states with MSPE of (2) ≤ 20×, (3) ≤ 5×, and (4) ≤ 2× that of the treatment state’s synthetic control MSPE [32, 38, 39]. Given the number of treatment states and outcomes, we will adjust for multiple comparisons using Benjamini-Hochberg adjustments [40].

As noted above, our primary analysis will produce 18 state-specific estimates of the effects of the four types of state laws of interest on opioid prescribing and chronic non-cancer pain treatment outcomes. The results of each state-specific quantitative model will then be interpreted with the help of the qualitative data collected for that state, e.g., results showing that a pill mill law reduced measures of high-risk opioid prescribing in state A but not in state B could be explained by qualitative data suggesting that state A engaged in significant implementation and enforcement efforts and state B did not. In a secondary analysis, we will explore the use of meta-regression [41–43] to quantitatively examine how differences in implementation and enforcement influence each of the four types of state laws effects on outcomes. Due to relatively small sample sizes (five treatment states with mandatory PDMP enrollment, mandatory PDMP query, and prescribing cap laws and three treatment states with pill mill laws), we consider this approach “exploratory.”

## Discussion

Through purposive selection of states that implemented a single law of interest and appropriate state-specific controls, as well as the use of study periods specific to each treatment state, the synthetic control design allows us to evaluate the independent effects of the opioid prescribing laws of interest on high-risk opioid prescribing and treatment of chronic non-cancer pain, overcoming methodological challenges related to the fact that many states have implemented multiple laws designed to reduce high-risk opioid prescribing at or around the same time.

A key element of our mixed-methods study is the use of qualitative data on state laws’ implementation and enforcement to inform quantitative model design and interpretation. The synthetic control method, which is designed to evaluate the outcomes of a single policy in a single state, is well-suited to this approach. State-specific study periods will be adjusted if qualitative interviews identify implementation or enforcement delays. Rather than producing an estimate of a given type of law’s average effects on outcomes across all states implementing that law, our analysis will produce state-specific estimates, allowing us to inform interpretation of results with insights about implementation and enforcement across states. If only one of the five states enacting a mandatory PDMP enrollment law had a strong implementation model (e.g., rigorous outreach/publicity, high awareness of law, mechanisms in place for assessing compliance), the results of a model that assumes constant effects across states—like the comparative interrupted time series models [44] often used to analyze laws’ effects—would likely show no effect of the law on outcomes, while our study design would instead lead us to conclude that this type of law may be effective if accompanied by significant implementation and enforcement efforts.

Our study has several limitations. The IQVIA claims data does not capture over-the-counter pain medications or include mail order prescriptions [35], which account for approximately 20% of all ≥ 90-day maintenance prescriptions filled [45]. Some patients in the IQVIA data will not have available data across all study years, but our research team has experience constructing continuous analytic cohorts using the IQVIA data to minimize bias resulting from patients dropping in and out of the sample [18, 19, 24]. Our use of administrative claims data does not allow us to determine the clinical appropriateness of pain treatments, and data on non-clinical pain treatments such as yoga are not available.

Despite widespread agreement that the opioid epidemic is driven by high rates of opioid prescribing by healthcare providers, little is known about the implementation, enforcement, and outcomes of policies designed to curb high-risk opioid prescribing practices. Our study assesses the independent effects of the four most common types of state laws designed to curb opioid prescribing on opioid prescribing patterns and chronic non-cancer pain treatment. Results will inform the dynamic policy environment in which numerous states pass, revise, implement, and enforce varied laws to address opioid prescribing each year.

## Abbreviations

PDMP: Prescription Drug Monitoring Program

## Footnotes

1 States with mandatory PDMP enrollment laws as of January 1, 2017: Alabama, Alaska, Arizona, Arkansas, California, Colorado, Connecticut, Delaware, Idaho, Illinois, Maine, Maryland, Massachusetts, Minnesota, Mississippi, Nevada, New Hampshire, New Mexico, Texas, Utah

2 States with mandatory PDMP query laws as of January 1, 2017: Arizona, Connecticut, Indiana, Kentucky, Louisiana, Maine, Massachusetts, New Hampshire, New Jersey, New Mexico, New York, Ohio, Oklahoma, Pennsylvania, Rhode Island, Tennessee, Vermont, Virginia, West Virginia, Wisconsin

3 States with opioid prescribing cap laws as of January 1, 2017: Connecticut, Maine, Massachusetts, New Hampshire, New York, Rhode Island, Vermont

4 States with pill mill laws as of January 1, 2017: Alabama, Florida, Georgia, Kentucky, Louisiana, Mississippi, Ohio, Tennessee, Texas, West Virginia, Wisconsin
